# A Case of Septic Arthritis Caused by Staphylococcus schleiferi

**DOI:** 10.7759/cureus.16669

**Published:** 2021-07-27

**Authors:** Nabin K C, Binav Shrestha, Sandhya Sharma, Baikuntha Chaulagai

**Affiliations:** 1 Internal Medicine, Interfaith Medical Center, Brooklyn, USA; 2 Internal Medicine, GetWell Medcare PC, Woodside, USA

**Keywords:** staphylococcal schleiferi, septic arthritis, septic emboli, misdiagnosed staphylococcal aureus, case report

## Abstract

Septic arthritis leads to significant hospital burden in the United States adult patient population. Bacteria are the leading cause of septic arthritis with *Staphylococcus aureus* being the most common. Of the staphylococcal species, *Staphylococcus ​​​​​​*​*schleiferi*, primarily found in carnivores, rarely causes septic arthritis. We here report the presentation, diagnosis, treatment, and discharge of a 39-year-old male with *S. schleiferi* septic arthritis. Due to biochemical similarities, *S. schleiferi* are commonly misidentified as *S. aureus*, and correct identification is increasingly relevant for the selection of appropriate therapy due to the rise in cases of multidrug-resistant microorganisms.

## Introduction

Septic arthritis, a serious type of joint infection, is responsible for a significant number of hospitalizations in the United States. One study suggests septic arthritis accounts for 0.01% of all adult emergency room visits from 2009-2012 in the United States, with the majority of patients requiring hospitalization (82%-84%) [1]. It is commonly caused by bacteria although other causative microorganisms have been reported [2]. Staphylococcal bacteria remain the leading cause of septic arthritis in adults [3] with *Staphylococcus schleiferi*, first reported in 1988 by Freney et al., being one example. *S. schleiferi* is a Gram-positive, facultatively anaerobic bacteria [4] commonly misidentified as S. aureus [5]. There has been case reports of *S. schleiferi* causing diabetic foot osteomyelitis, bacteremia, pacemaker infection and vertebral osteomyelitis in humans [6,7]. Here we present a rare case of septic arthritis caused by *S. schleiferi*.

## Case presentation

​​​​​A 39-year-old male with a history of diabetes mellitus was admitted complaining of generalized body ache and fatigue for a week. The patient reported having bilateral arm pain; it was a dull 8/10 on a pain scale and associated with limited range of motion in both shoulder joints. He denied any trauma to the joints. The patient reported non-bloody loose stools during the same period but no fever, chills, abdominal pain, cough, sore throat, urinary symptoms, limb weakness, or rash. He denied any recent travel, being in contact with anyone with similar symptoms, and positive testing for COVID-19. The patient was sexually active with a single female partner.

Triage vital signs were significant for temperature of 102.6°F and heart rate of 152 beats per minute while all other signs were unremarkable. On physical examination, the patient was found to have a soft non-distended abdomen with mild tenderness on deep palpation but no organomegaly and normal bowel sound on auscultation. The left testicle was enlarged with mild tenderness but returned a negative transillumination test. A musculoskeletal examination was significant for mild tenderness in both shoulders with relatively more swelling in the right joint. The patient’s motor strength in both upper extremities was 3/5, likely due to pain with no focal neurological deficit. The rest of the physical examination was non-significant.

**Figure 1:**
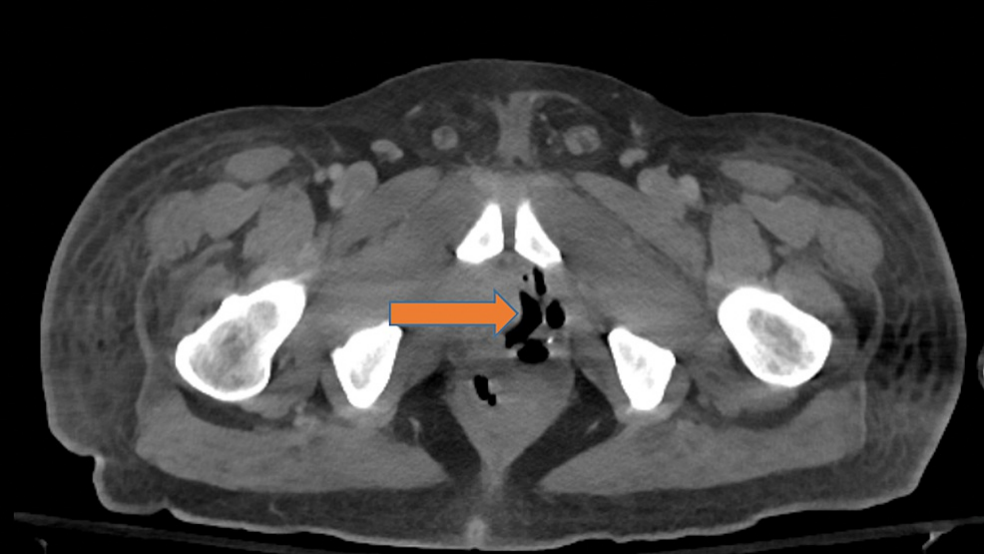
CT abdomen and pelvis showing enlargement and heterogeneity of the left seminal vesicles, with soft tissue fullness and obliteration of the fat planes between the rectum, prostate gland, and seminal vesicles(shown by orange arrow). These findings are concerning for gas-forming infectious process.

**Figure 2:**
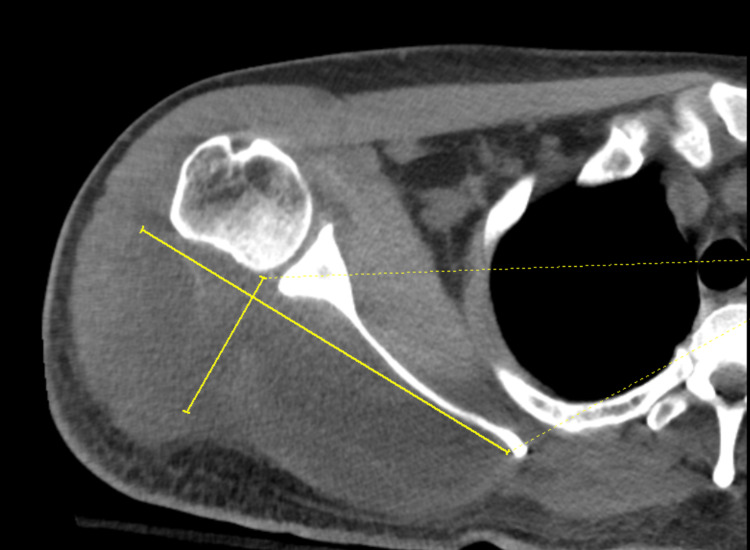
CT of the right shoulder showing a hypodense intramuscular collection (yellow lines marking length and breadth of the collection) with no signs of gas, concerning for intramuscular hematoma versus infection specifically in the infraspinatus muscle.

**Figure 3:**
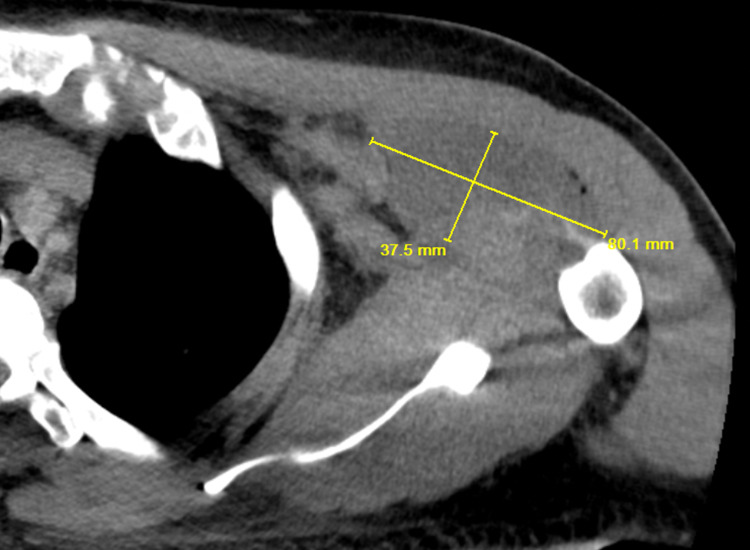
CT of left shoulder joint showing a hypodense collection (yellow lines showing dimensions, length and breadth of the collection) with multiple foci of air concerning for intramuscular hematoma/abscess which measures 8.0 x 3.8 cm in the anterior left shoulder, specifically the musculature of the anterior deltoid and pectoralis major/minor.

The patient was initially admitted for sepsis likely due to urogenital infection with the possibility of epididymo-orchitis versus necrotizing infection in the perineum versus septic arthritis of the bilateral shoulder joints: ultrasound of the scrotum was suggestive of left epididymo-orchitis; CT of the abdomen and pelvis (Figure 1) had suggested a gas-forming infectious process in the perineum; and imaging of the shoulders had shown a moderate amount of fluid within the subacromial-subdeltoid bursae as well as within the joint(Figure 2 and Figure 3)*.* Blood cultures were sent, and CT-guided aspiration of the joints with purulent fluid study was performed. The patient was started initially on broad-spectrum antibiotics (vancomycin and meropenem) but later switched first to nafcillin and then to cefazolin and clindamycin as the blood cultures grew methicillin-sensitive *S. aureus* (MSSA) bacteremia and the aspirated fluid was positive for *S. schleiferi*. Both transthoracic and transesophageal echocardiograms were negative for vegetations. An HIV test was negative and immunoglobulin levels for IgA, IgM, IgE, and IgG were all normal, thus ruling out common immunodeficiency diseases. Inflammatory markers were high with an ESR of 171, CRP of 401, and C3 of 239. Rheumatoid factor as well as antimitochondrial, anti-smooth muscle, antinuclear, and other rheumatologic antibodies were negative.

**Figure 4:**
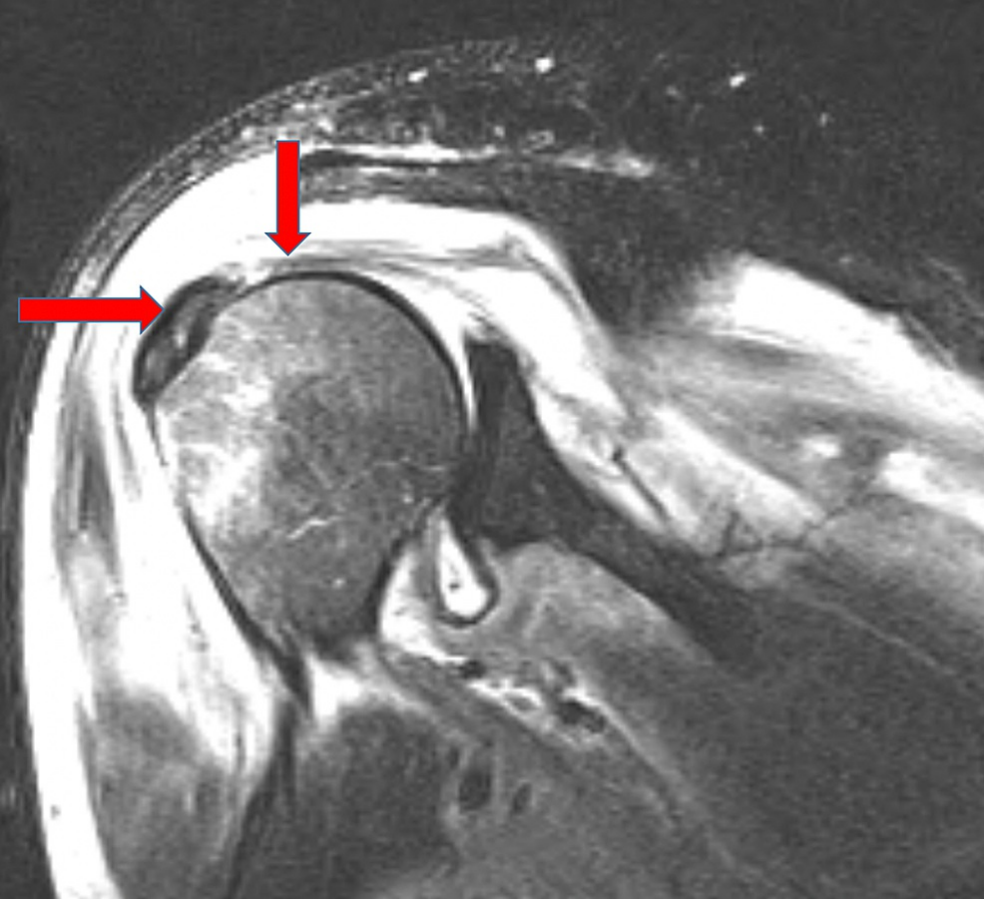
MRI of right shoulder: Moderate of fluid (as shown by the red arrows) within the subacromial-subdeltoid bursae as well as within the joint which represents septic arthritis. Diffuse edema especially within the infraspinatus muscle may represent infectious myositis or reactive changes.

**Figure 5:**
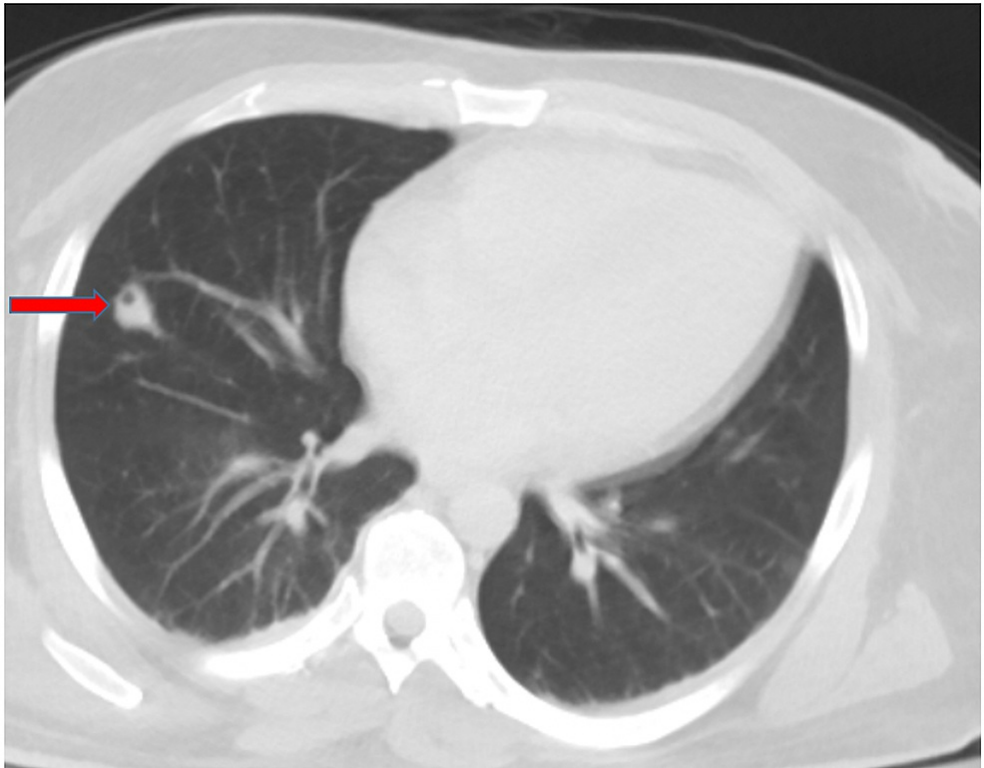
CT pulmonary angiography with septic embolus in the right lung as shown by the red arrow.

One week following the resolution of the effusion, the patient complained of bilateral shoulder pain and associated limitation of motion. A shoulder MRI was conducted which indicated inflammation of the muscle and ruled out abscess or osteomyelitis as shown in Figure 4. The patient’s pain and the swelling improved with administration of NSAIDs, and his inflammatory markers normalized. During the course of his hospitalization, the patient was afebrile. On day 13, however, he developed acute shortness of breath and tachycardia. A CT chest angiogram was performed as shown in Figure 5, which was suggestive of a septic embolus in the right lung, likely the disseminated MSSA bacteremia. A heparin drip was initiated, then switched to low molecular heparin, and ultimately bridging to oral NOAC.

As the patient’s symptoms were resolving, he was assessed by the physiatrist and discharged from hospital to subacute rehabilitation.

## Discussion

Septic arthritis is an infection of the joints and is commonly caused by bacteria although other causative microorganisms have been reported [2]. Though typically monoarticular, oligoarticular septic arthritis is not uncommon, and infectious arthritis usually follows hematogenous seeding to the predisposed joints [3]. One study reports that risk factors for developing septic arthritis are age ≥80 years, diabetes mellitus, rheumatoid arthritis, hip and/or knee prosthesis, joint surgery, and skin infection [8]. Staphylococcal bacteria are the leading cause of septic arthritis in adults [3]. *Staphylococcus schleiferi* is one such organism, first reported in 1988 by Freney et al., and commonly found in carnivores and occasionally transmitted from pets [4]. As for the case discussed, he had diabetes mellitus as the single risk factor and had no history suggestive of interaction with pets.

*S. schleiferi* is a facultatively anaerobic Gram-positive cocci which forms round, nonpigmented colonies that are beta-hemolytic on sheep blood agar [9]. Several biochemical reactions differentiate *S. schleiferi* from other staphylococcal species in that it is characteristically pyrrolidonyl arylamidase and alkaline phosphatase positive, ornithine decarboxylase negative, and susceptible to novobiocin and polymyxin B [9]. *S. schleiferi* can be easily confused with *S. aureus* in a typical clinical laboratory since both possess heat-stable DNase and promote clotting formation [5]. Coagulase-positive gram-positive cocci in clusters or slide agglutination reaction may be mistaken as *S. aureus*. *S. schleiferi* does not acidify sucrose, maltose or mannitol as oppose to *S. aureus* and reacts positively for PYR. Essentially, matrix-assisted laser desorption ionization-time of flight (MALDI-TOF) mass spectrometry (MS) can accurately separate *S. schleiferi* from other coagulase-positive staphylococcal organisms [7].

Cases of *S. schleiferi* infection in humans have been reported in the literature including skin, soft tissue, and device infections, osteomyelitis, endocarditis, and bacteremia [10,11]. A case of a 60-year-old female with bacteremia and vertebral osteomyelitis was reported by Yarbrough et al. [7]. The patient reported by Yarbrough et al. was treated with intravenous vancomycin and later de-escalated to ceftriaxone for 6 weeks with complete resolution and no adverse clinical progression [7]. Similarly a rare case of *S. schleiferi* causing diabetic foot osteomyelitis and bacteremia was reported by Nguyen and team in an immunocompromised patient [6]. The patient was initially reported to have grown *Staphylococcal epidermidis* which was later rectified as *S. schleiferi* on day 5 and treated with broad-spectrum antibiotics later de-escalated to piperacillin-tazobactam and subsequently to ceftriaxone for a total duration of six weeks [6]. The case we reported was treated initially with vancomycin and meropenem, later de-escalated to cefazolin and clindamycin based on culture and sensitivity report.

As the number of cases in human due to *S. schleiferi* surges the importance of correctly identifying the organism becomes more significant.

## Conclusions

Septic arthritis caused by *S. schleiferi* is a rarely reported case. *S. schleiferi* is easily confused with S. aureus as both possess heat-labile DNase and promote clotting formation. It demands strong suspicion and specific biochemical and microbiological tests to identify it. Correct identification of such microorganisms helps drive appropriate antimicrobial use which is a crucial practice in preventing multidrug-resistant organisms.
